# Different adsorption-degradation behavior of methylene blue and Congo red in nanoceria/H_2_O_2_ system under alkaline conditions

**DOI:** 10.1038/s41598-018-36794-2

**Published:** 2019-03-21

**Authors:** Xiaoshu Wei, Yi Wang, Yuqian Feng, Xiaomin Xie, Xiaofeng Li, Sen Yang

**Affiliations:** Beijing Key Laboratory of Farmland Soil Pollution Prevention and Remediation, College of Resources and Environmental Sciences, China Agricultural University, Beijing, 100193 China

## Abstract

The Fenton-like activity of nanoceria has attracted intensive attention for wastewater treatment in recent years. During the Fenton-like reaction, the adsorption of organic pollutants on catalyst surface plays a key role in their degradation. In this work, the adsorption-degradation of methylene blue (MB) and Congo red (CR) in nanoceria/H_2_O_2_ system was investigated under alkaline conditions. The MB exhibited weak adsorption on nanoceria surface via electrostatic attraction, while strong Lewis acid–base interactions between CR and cerium ions was observed. Moreover, the adsorption of MB was enhanced in the presence of H_2_O_2_ by the formation of surface peroxide species, but an adsorption competition existed between H_2_O_2_ and CR. With more Ce^3+^, CeO_2_ nanorods could degrade CR efficiently as Fenton-like catalyst. But the degradation of MB catalyzed by ceria was much lower than that of CR in the presence of H_2_O_2_.

## Introduction

Recently, many studies have suggested that ceria nanoparticles (nanoceria) can act as a Fenton-like catalyst, based on observations of the oxidation reaction of organic compounds in the presence of H_2_O_2_^1–3^. The Fenton-like activity of nanoceria has been ascribed to some active oxidative species that can be generated during the catalytic decomposition of H_2_O_2_^4–6^. In 2008, Heckert *et al*.^4^ first reported that a cerous salt solution could catalyze the decomposition of H_2_O_2_ and generate HO∙ through a Fenton-like reaction. Later, the formation of HO· was confirmed in nanoceria/H_2_O_2_ system^7^. On the other hand, Chen and co-workers^8–10^ revealed that H_2_O_2_ could react with cerium ions on the surface of nanoceria and form stable brown peroxide species, which would induce an intermolecular rearrangement with the adjacent adsorbed organic compounds to achieve the degradation of organics or generate HO∙ through a homolytic cleavage of the O–O bond to attack the neighboring adsorbed organics^5^. Now, it is recognized that the catalytic activity of nanoceria relies closely on the redox cycle between Ce^3+^ and Ce^4+^, and a higher level of Ce^3+^ and defects can generate more active oxidative species and exhibit better activity toward the oxidative degradation of organic compounds^11,12^.

The oxidation reaction of organic compounds in nanoceria/H_2_O_2_ system generally occurs on the ceria surface; therefore, the adsorption of organics plays an important role in their degradation^10,13^. Previous studies have shown that nanoceria exhibits high degradation activity for the adsorbable organic compounds such as orange II, methyl orange, salicylic acid^8,14^; however, the degradation of weakly adsorbed organic compounds such as rhodamine B, rhodamine 6 G and catechol hardly occurs^6,8^. Actually, the adsorption of organics depends on the structures of organic compounds, the surface chemistry of adsorbent and the solution conditions^15,16^. According to adsorbate–adsorbent interactions, the adsorption can be classified as chemical adsorption and physical adsorption^16^. Chemical adsorption means the formation of strong chemical associations between adsorbate and adsorbent; therefore, chemical adsorption is usually irreversible. Physical adsorption is reversible and the main physical forces controlling adsorption are van der Waals forces, hydrogen bonds and polarity^17^. The presence of H_2_O_2_ also influences the adsorption of organic compounds over nanoceria surface. For example, the adsorption competition between H_2_O_2_ and orange II has been observed^5,13^, and the degradation of orange II was inhibited by over-complexation of H_2_O_2_ with CeO_2_^10,18^. Because of the importance of adsorption in Fenton-like processes, it is highly desirable to investigate the adsorption behavior of organic compounds in nanoceria/H_2_O_2_ system and their effect on the degradation of organic compounds.

Today, more than 100,000 commercial dyes with different chemical structures are widely used for printing and dyeing and a portion is discharged with wastewater^19,20^. Several methods such as advanced oxidation and adsorption are used to decolorize dye wastewater^16,21^. Fenton-like reaction is found to be efficient for the removal of organic pollutants from wastewater^21–23^. Generally, pH has an important effect on the efficiency of Fenton-like catalysts such as Cu-based bimetallic oxides, Fe_3_O_4_@cellulose aerogel nanocomposite and Mn-doped BiFeO_3_ nanoparticles^24–26^. Some studies showed that the optimum working condition is acidic condition, while others reported that some catalysts could efficiently decompose H_2_O_2_ even at near-neutral or neutral conditions^27,28^. These researches mainly focus on the acidic and neutral pH conditions. As we know, Aneggi *et al*.^2^ firstly reported that ceria and ceria-zirconia solid solutions could be effectively used for the treatment of landfill leachate at pH 9.0. Actually, the wastewater generated during printing and dyeing is characterized by a high pH value^29^. Then, methylene blue (MB), one of the major thiazine dyes, was chosen as a representative for cationic dyes, and Congo red (CR), one of the major azo dyes was used as a model for anionic dyes. CeO_2_ nanocubes and nanorods were prepared by a hydrothermal method and were used as Fenton-like catalysts for the degradation of dyes in the presence of H_2_O_2_. The adsorption of dyes on ceria surface in the presence or absence of H_2_O_2_ was investigated to better understand their degradation in nanoceria/H_2_O_2_ system under alkaline conditions.

## Results and Discussion

### Characterization of nanoceria

**Figure 1:**
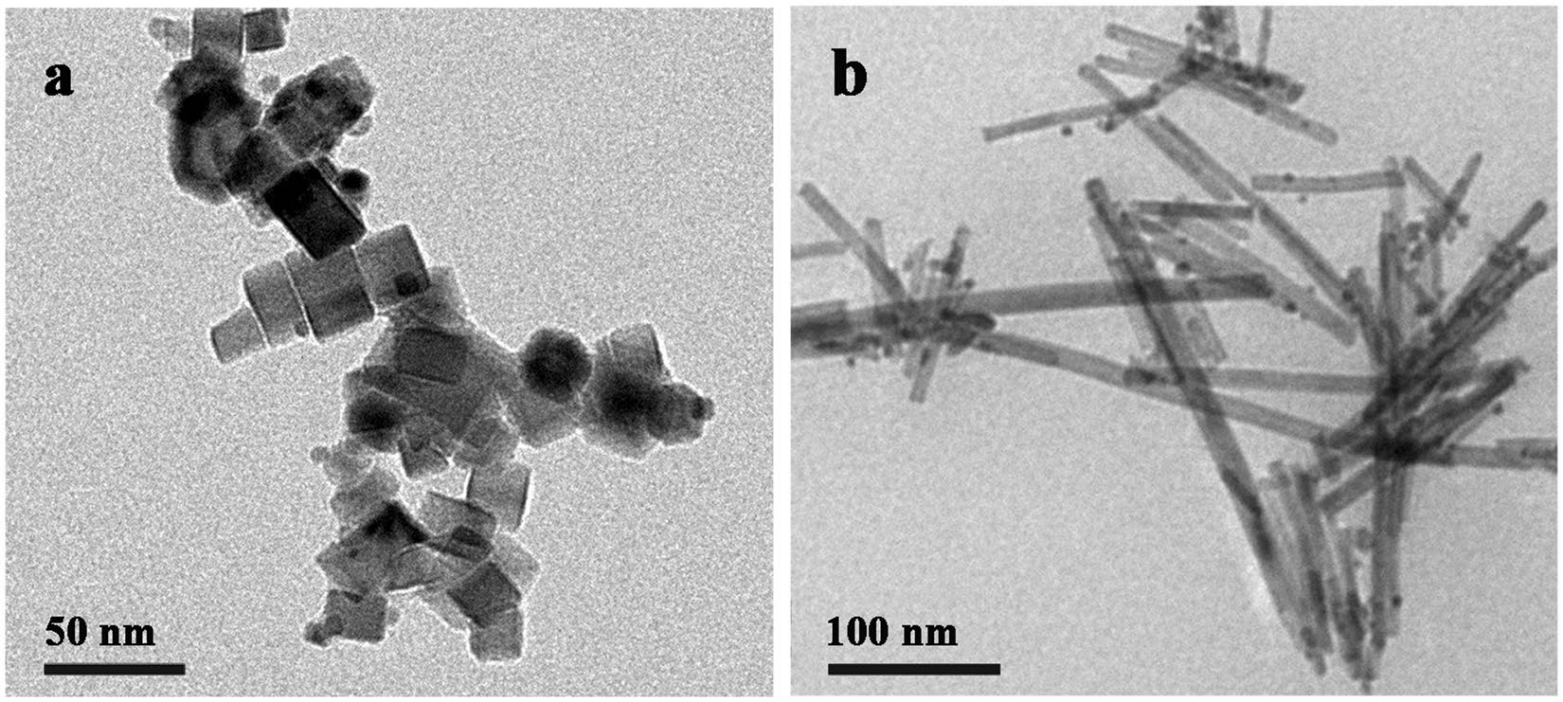
TEM images of CeO_2_ nanocubes (**a**) and nanorods (**b**).

Nanoceria was synthesized through a hydrothermal process. The morphologies and sizes of nanoceria were studied by transmission electron microscopy (TEM). As in our previous study^30,31^, the CeO_2_ nanocubes had uniform cubic shapes with a size of 20–30 nm (Fig. 1a), while the CeO_2_ nanorods had diameters of approximately 15–20 nm and lengths of 100–200 nm (Fig. 1b). Dynamic light scattering analysis was performed to detect the agglomeration size of nanoceria in pH 9.0 of aqueous solution. As shown in Fig. S1, both of nanocubes and nanorods are inclined to agglomerate into larger particles with similar size.

**Figure 2:**
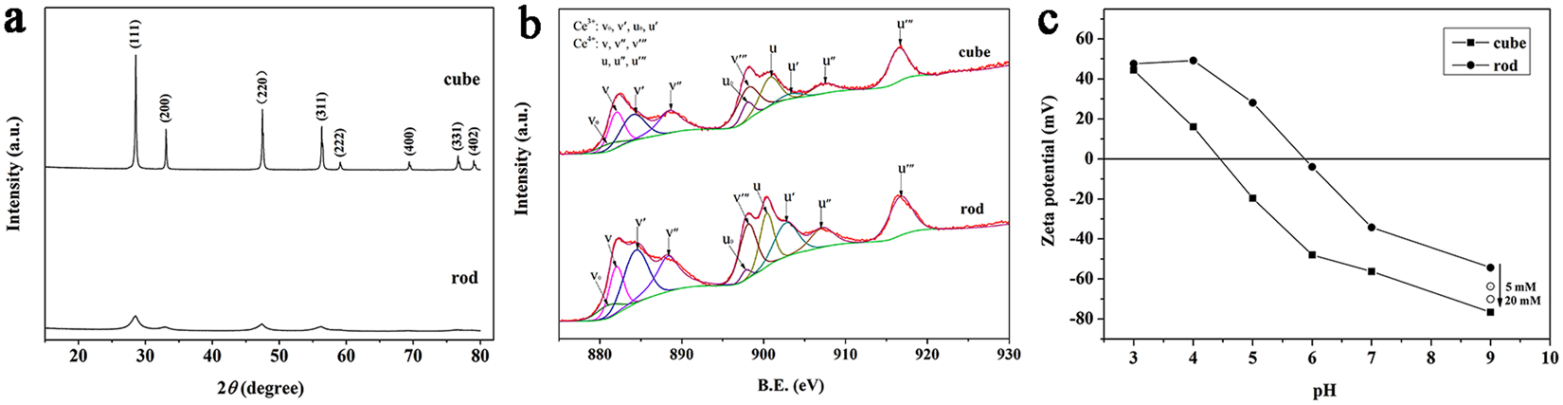
XRD patterns (**a**), Ce3d XPS spectra (**b**) and the zeta potential as a function of pH of CeO_2_ nanorods and nanocubes (**c**).

The phase purity and crystal structure of CeO_2_ nanocubes and nanorods were identified by XRD (Fig. 2a). All the diffraction peaks of both samples can be indexed to the pure fluorite structure of CeO_2_ (JCPDS 34–0394). Meanwhile, the nanocubes exhibited sharper XRD diffraction peaks than those of the nanorods, indicating a higher crystallinity and bigger crystallite size of the nanocubes^32^. The surface chemistry of nanoceria was analyzed by XPS. The XPS survey scan is given in Fig. S2. The possibility of appearance of sodium on the surface of nanoceria can be ruled out because no peaks belonging to sodium were detected. The Ce(*3d*) XPS spectra are shown in Fig. 2b. Peak v_o_, v’, u_o_ and u’ belong to Ce^3+^ species, while v, v′′, v′′′, u, u′′ and u′′′ are derived from Ce^4+^. The relative concentration of Ce^3+^ on the surface of nanoceria was calculated as follows^33^:

1 $$[C{e}^{3+}]=\frac{{A}_{v0}+{A}_{v^{\prime} }+{A}_{u0}+{A}_{u^{\prime} }}{{A}_{v0}+{A}_{v^{\prime} }+{A}_{u0}+{A}_{u^{\prime} }+{A}_{v}+{A}_{v^{\prime\prime} }+{A}_{v\prime\prime\prime }+{A}_{u}+{A}_{u^{\prime\prime} }+{A}_{u\prime\prime\prime }}\times 100 \% $$

where A_i_ is the integrated area of peak ‘i’.

The relative concentration of Ce^3+^ on the surface of nanorods was 42.9%, which was much larger than that of nanocubes (36.5%). The O(*1 s*) spectra for both samples are shown in Fig. S3. The peak with a binding energy of around 529 eV can be ascribed to the lattice oxygen species of bulk CeO_2_ (O_lat_); the peak with a binding energy of around 531 eV is attributed to surface chemisorbed oxygen (O_sur_) and the peak at around 533 eV is assigned to molecular water adsorbed on the surface^34–36^. According to the literature^34–36^, the relative concentration of O_sur_ is estimated from the relative areas of peaks. The ratios of O_sur_/(O_lat_ + O_sur_) are approximately 50.6% and 59.4% for nanocubes and nanorods, respectively. The higher proportion of chemisorbed oxygen in nanorods may due to their higher Ce^3+^ concentration. Because Ce^3+^ in nanoceria can generate oxygen vacancies and then facilitate oxygen adsorption^37^.

The zeta potential of nanoceria was determined as a function of pH, and the isoelectric points (PI) of nanocubes and nanorods was at approximately pH 4.5 and 5.8, respectively (Fig. 2c). The specific surface areas of nanoceria were measured by nitrogen gas adsorption/desorption isotherm and calculated by BET method. The nanorods exhibit a larger surface area (89.9 m^2^ g^−1^) than that of nanocubes (39.7 m^2^ g^−1^).

### Adsorption of MB and CR on nanoceria in the absence of H_2_O_2_

To understand the decolorization of MB and CR in nanoceria/H_2_O_2_ system, the adsorption of dyes on nanoceria surface was surveyed in the absence of H_2_O_2_ at first, and the adsorbed amount of dye was determined after 30 min of equilibrium time. The amount of MB adsorbed on CeO_2_ nanorods and nanocubes was only 0.01 mg m^−2^ and 0.11 mg m^−2^, respectively. The results showed that the adsorption of MB on CeO_2_ surface was very weak. Interestingly, a large amount of negatively charged CR was adsorbed: 0.78 mg m^−2^ on the nanorods surface and 0.39 mg m^−2^ on the nanocubes surface (Fig. S4a).

The different adsorption behavior could be possibly explained by the different structures of MB and CR. At pH 9.0, nanocubes and nanorods are negatively charged (Fig. 2c), and MB is positively charged. Under these conditions, MB adsorption on the nanoceria surface mainly takes place through electrostatic attraction, and the higher adsorption capacity of nanocubes may be owing to the higher negative charge^38^. Similar adsorption behavior has also been observed for cationic dye rhodamine B (RhB) and Orange II, which were adsorbed by CeO_2_ through strong electrostatic attraction^13,39^. However, the electrostatic repulsion between CR and nanoceria means that the strong adsorption of CR was not based on the electrostatic interaction. *Ex situ* FT-IR data indicated that acid orange 7 (AO7), an azo dye, could be adsorbed on nanoceria surface via a Lewis acid–base reaction between cerium ions and the oxygen atoms of sulfonate group of azo dye^40^. Srilakshmi^41^ reported that Ag-modified calcium hydroxyapatite (CaHAp) exhibited high adsorption capacity for CR adsorption because of its high Lewis acidity. Thus, we speculated that CR adsorption was based on the Lewis acid−base reaction, and more Ce^3+^ on nanoceria surface resulted in a higher adsorption capacity.

To further investigate the adsorption mechanism, desorption study was performed using HCl aqueous solution with pH of 3.0 as the eluting agent. For the desorption study, the dye-loaded nanoceria was isolated from pH 9.0 suspension and added into the HCl aqueous solution. A quick desorption of MB occurred. The colorless acid aqueous solution became blue almost immediately after the introduction of MB-loaded nanoceria. The occurrence of desorption could be explained by electrostatic repulsion between MB and nanoceria because the surface of nanoceria is positively charged at pH 3.0. Therefore, we can conclude that both nanocubes and nanorods could adsorb MB through electrostatic adsorption when the pH of solution was higher than their PI and the adsorption of MB was reversible. However, the desorption of CR did not occur and the adsorbed CR retained its red color in the HCl aqueous solution, even though free CR is a pH indicator and should turn blue at pH 3.0. The strong adsorption of CR on nanoceria further indicated that the interaction between CR and ceria surface could be owing to the strong Lewis acid–base interactions.

### Decomposition of H_2_O_2_ over CeO_2_

**Figure 3:**
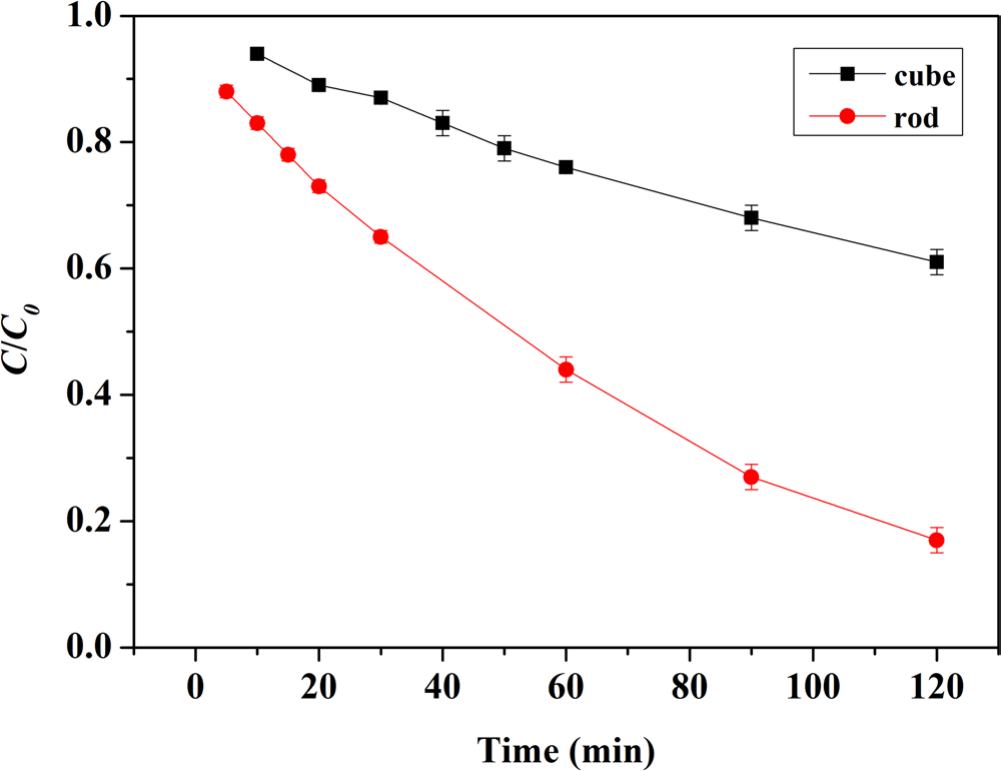
H_2_O_2_ decomposition over CeO_2_ nanorods and nanocubes (1.0 g L^−1^ CeO_2_, 20 mM H_2_O_2_, 25 °C).

The catalytic H_2_O_2_ decomposition over CeO_2_ nanorods and nanocubes was investigated at 25 °C. As shown in Fig. 3, 82.7% of 20 mM H_2_O_2_ was decomposed after 120 min in the presence of nanorods, while only 38.8% in the presence of nanocubes under the same conditions. Generally, the catalytic activity of nanoceria is directly related to its surface chemical states and specific surface area^11^. The high concentration of Ce^3+^ facilitates the adsorption and decomposition of H_2_O_2_^4,5,42^. Hence, the high activity of nanorods was owing to the presence of more Ce^3+^, as proved by XPS analysis.

To probe the oxidative species during the catalytic decomposition of H_2_O_2_ over nanoceria, EPR spectroscopy measurement was performed using DMPO as a spin trap. As shown in Fig. S5, a typical signal of DMPO-OH adducts (1:2:2:1 quartet) was detected for a suspension of nanorods upon the addition of H_2_O_2_, which suggested the generation of HO· in the catalytic decomposition of H_2_O_2_. Similar results were obtained for a suspension of nanocubes, except the low intensity of the DMPO-OH peaks. Fig. S6 showed the Raman spectra of CeO_2_ nanorods before and after H_2_O_2_ treatment at pH 9.0. The appearance of band at 830 cm^−1^ indicates the presence of the *η*^2^-peroxide (O_2_^2-^) species on the surface of CeO_2_ nanorods after H_2_O_2_ treatment^8,43,44^. These results suggest that the presence of HO· and peroxide-like intermediates in nanoceria/H_2_O_2_ system at pH 9.0. Hamoud *et al*.^13^ suggested the relationship between surface Ce(IV) peroxo species and HO· radicals production:

2 $$C{e}^{3+}+\frac{3}{2}{H}_{2}{O}_{2}\to C{e}^{4+}{({O}_{2})}^{2-}+{H}_{2}O+{H}^{+}$$

3 $$C{e}^{4+}{({O}_{2})}^{2-}+2{H}^{+}\to C{e}^{4+}+2HO\cdot $$

### Decolorization of MB in nanoceria/H_2_O_2_ system

#### Effect of H_2_O_2_ concentration on decolorization of MB

Pre-experiments showed that little change in MB concentration was observed with H_2_O_2_, however, upon introduction of H_2_O_2_ into suspension of nanoceria with MB, the color of nanocubes and nanorods became deep blue and green in 5–10 min, respectively (Fig. S7). Obviously, the adsorption of MB on nanoceria was enhanced in the presence of H_2_O_2_. And the decolorization of MB in nanoceria/H_2_O_2_ system can be ascribed to both adsorption and oxidative degradation. Then the total amount of MB (labeled as MB_t_) in suspension was divided into three parts: (1) free MB (labeled as MB_f_) in supernatant; (2) adsorbed MB (labeled as MB_a_) on nanoceria surface; and (3) degraded MB (labeled as MB_d_) in nanoceria/H_2_O_2_ system, which was determined by the difference in the amount of MB_t_, MB_f_ and MB_a_:

4 $$M{B}_{d}=M{B}_{t}-M{B}_{f}-M{B}_{a}$$

**Figure 4:**
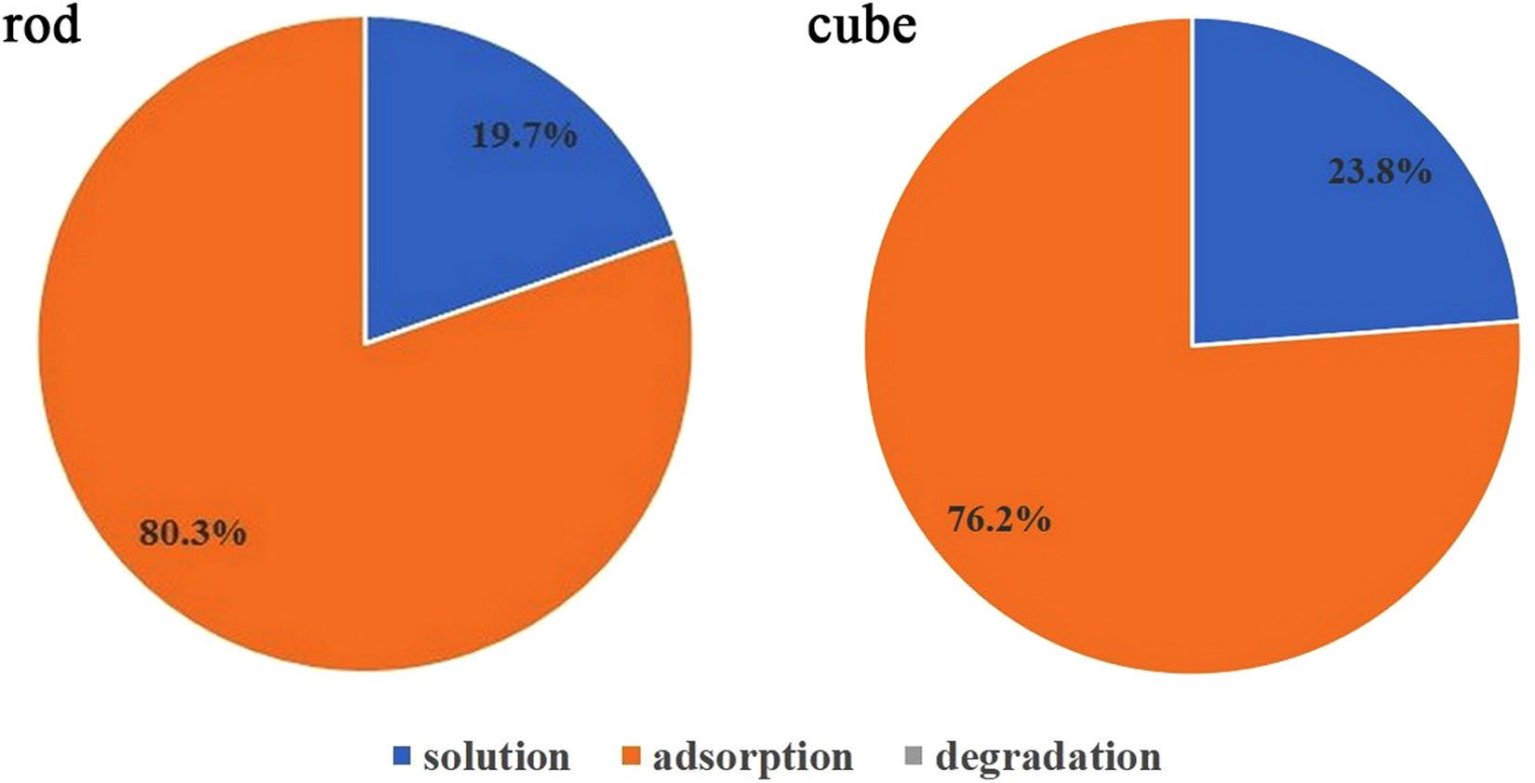
Distribution of MB in nanoceria/H_2_O_2_ system at 30 min.

When the concentration of H_2_O_2_ was 20 mM, the decolorization of MB after 30 min in both nanoceria/H_2_O_2_ systems was investigated. In the case of nanorods, 80.3% of MB was decolorized; in the case of nanocubes, the decolorization was 76.2% (Fig. 4). However, when the nanoceria was isolated from both systems and added into pH 3.0 HCl aqueous solution, desorption occurred and the amount of desorbed MB was almost equal to the amount of decolorization. The results clearly suggested that the decolorization of MB in nanoceria/H_2_O_2_ system was mainly due to adsorption rather than degradation at 30 min. Meanwhile, the enhanced adsorption of MB on nanoceria surface caused by the presence of H_2_O_2_ was reversible and could be totally desorbed using pH 3.0 HCl aqueous solution as the eluting agent.

**Figure 5:**
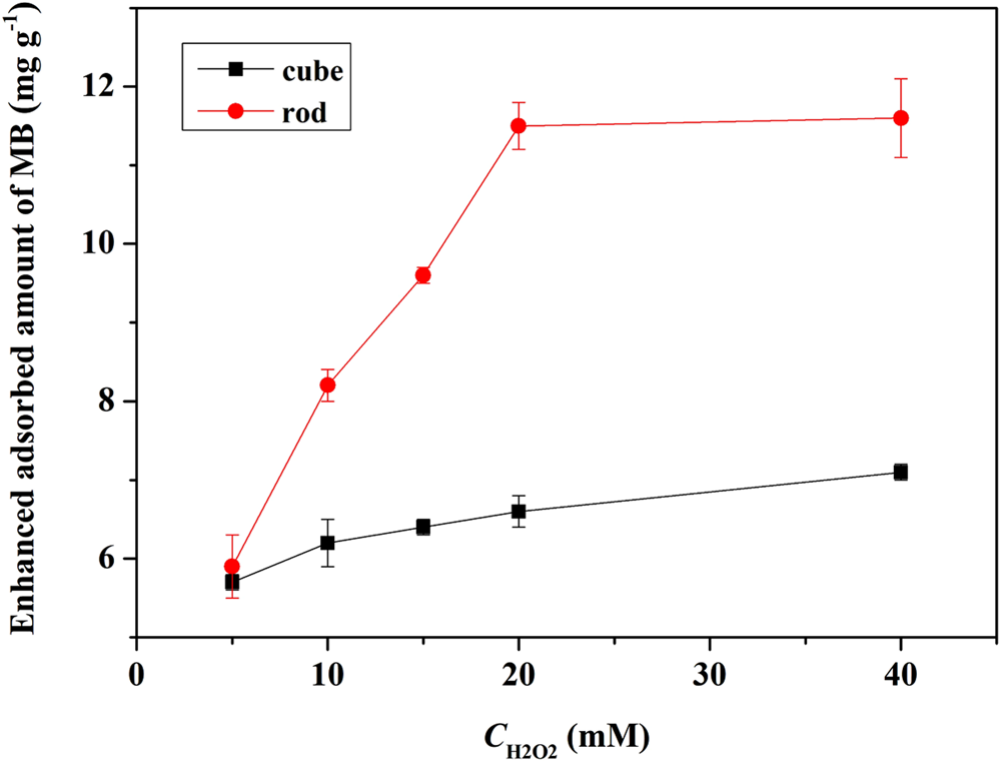
The effect of H_2_O_2_ concentration on the adsorption of MB on nanoceria surface (15 mg L^−1^ MB, 1.0 g L^−1^ CeO_2_, 25 °C, pH 9.0).

The influence of H_2_O_2_ concentration on MB adsorption on nanoceria surface was further investigated. The contact time was limited to 30 min to avoid the interference of degradation of MB. As shown in Fig. 5, upon the introduction of 5 mM H_2_O_2_, the adsorbed amount of MB on CeO_2_ nanorods and nanocubes increased by 6.6 and 1.3 times in 30 min, respectively. In the case of nanorods, the adsorbed amount of MB increased with increasing H_2_O_2_ concentration from 5 mM to 20 mM and then remained almost unchanged. However, further increase of the H_2_O_2_ concentration did not have a significant influence on the adsorption of MB in the presence of nanocubes.

The effect of H_2_O_2_ on the zeta potential of nanoceria at pH 9.0 was investigated. It is clear that the addition of H_2_O_2_ made the zeta potential of CeO_2_ nanorods more negative, and the absolute value of the zeta potential increased from 5 mM to 20 mM of H_2_O_2_ (Fig. 2c). However, the zeta-potential of CeO_2_ nanocubes did not change obviously in the presence of H_2_O_2_ (data not show). The increased negative zeta-potential of CeO_2_ nanorods may be attributed to the formation of negative surface peroxide species via Ce^3+^–H_2_O_2_ interactions^43,45^. The generation of surface peroxide species increased with increasing H_2_O_2_ concentration until the Ce^3+^ sites of nanoceria surface were completely occupied^5,13^. The unchanged zeta-potential of CeO_2_ nanocubes may be explained by the presence of less Ce^3+^ sites and higher original zeta-potential. Because the adsorption of MB on nanoceria surface is via electrostatic adsorption, we speculate that the negatively charged surface peroxide species on the surface of nanoceria may be an important reason for the enhanced adsorption of MB on nanoceria, especially on the nanorods.

#### Effect of contact time on MB decolorization

**Figure 6:**
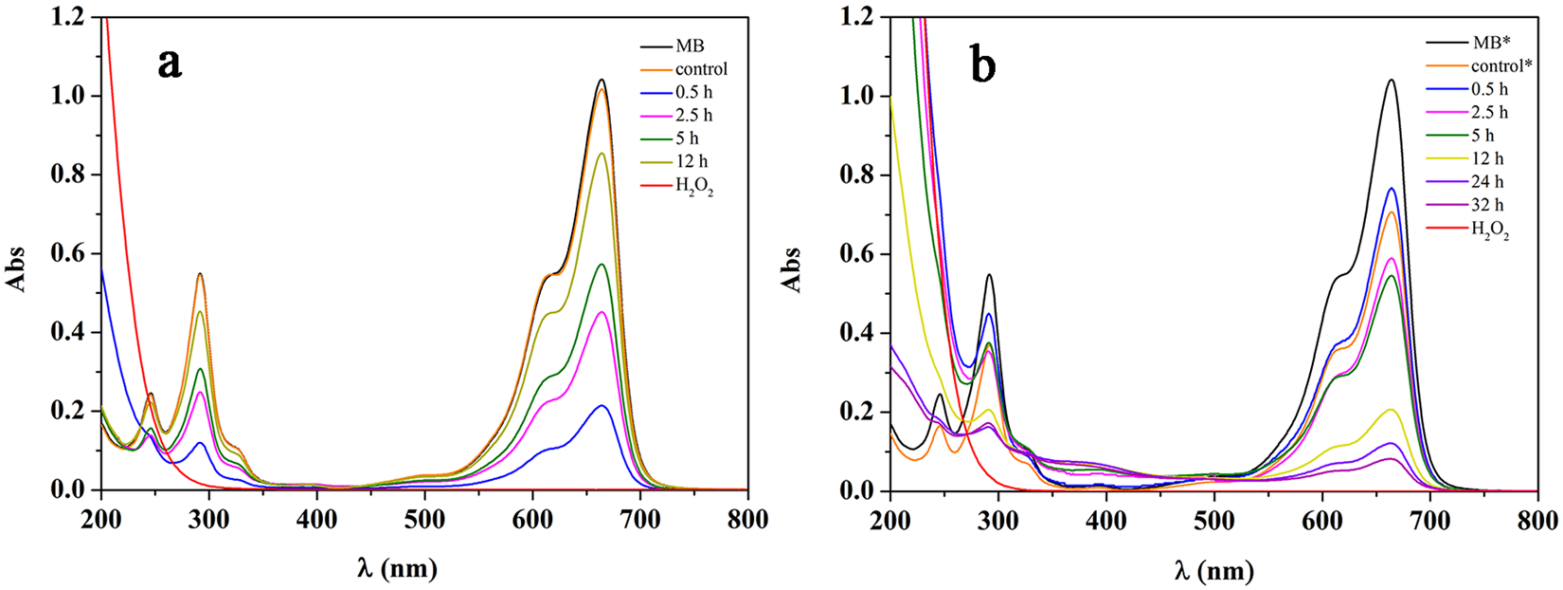
Effect of contact time on MB decolorization in nanoceria/H_2_O_2_ system (15 mg L^−1^ MB, 1.0 g L^−1^ CeO_2_, 20 mM H_2_O_2_). (**a**) CeO_2_ nanorods/H_2_O_2_ system (The samples were diluted 1:2 in pH 9.0 aqueous solution before tested). (**b**) CeO_2_ nanocubes/H_2_O_2_ system (the samples marked * were diluted 1:2 in pH 9.0 aqueous solution before tested).

To further investigate the catalytic oxidation of MB in nanoceria/H_2_O_2_ system, the effect of contact time on the decolorization of MB was investigated. Figure 6a shows the UV–vis spectral changes of the supernatant as a function of time. Upon the addition of H_2_O_2_ to the suspension of nanorods/MB, the characteristic band (centered at 664 nm) of MB decreased immediately, after which it continued to decrease up to 30 min, and then began to increase up to 12 h. The UV–vis spectra showed that the concentration of MB in supernatant had a special change: it first decreased and then increased.

**Figure 7:**
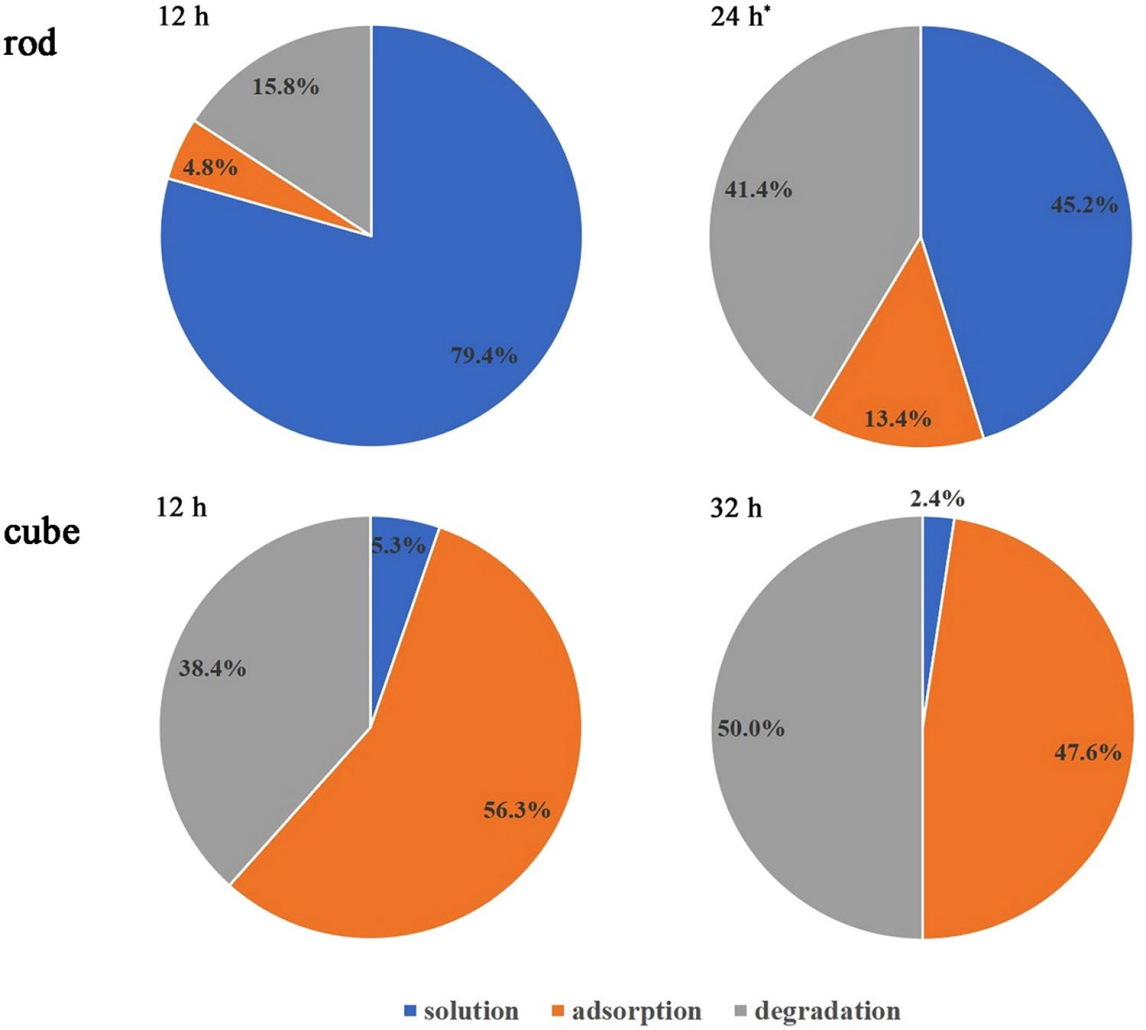
Distribution of MB in nanoceria/H_2_O_2_ system (*: the addition of H_2_O_2_ was repeated at 12 h).

To understand the decolorization of MB in CeO_2_ nanorods/H_2_O_2_ system, the distribution of MB in suspension at 12 h was analyzed and is shown in Fig. 7; most of MB was dissolved in supernatant, 15.8% was degraded and only 4.8% was adsorbed. These results indicated that MB could be degraded in suspension of nanocubes/H_2_O_2_ at pH 9.0, although the rate of degradation was rather slow. The special change of MB concentration in supernatant was the result of adsorption, desorption and degradation. But the change of MB concentration in supernatant was mainly determined by adsorption and desorption because the degradation rate of MB was low. During the 12 h long MB degradation reaction, adsorption dominated and the content of MB in supernatant quickly decreased during the first 30 min because of the formation of a large amount of surface peroxide species, and then MB was desorbed from nanoceria in the next 11.5 h due to the decomposition of surface peroxide species with long contact time. That was in accord with the remaining H_2_O_2_ concentration in system. The 240–290 nm spectral range, which corresponds to the H_2_O_2_ concentration, showed a continual decrease in absorbance with increasing reaction time (Fig. 6a).

The slow oxidative process of MB was likely due to their stability and difficult degradation. Meanwhile, few surface peroxide species on nanorods, which was caused by the high decomposition activity for H_2_O_2_, was also one important reason. To further confirm this assumption, we repeated this experiment and added H_2_O_2_ into the system again at 12 h (the final H_2_O_2_ concentration at 12 h was still 20 mM). As expected, the proportion of degraded and adsorbed MB increased to 41.4% and 13.4%, respectively (Fig. 7).

However, a different phenomenon was observed in nanocubes/H_2_O_2_ system. The absorbance band at 664 nm decreased promptly upon the addition of H_2_O_2_, after which it continued to decrease gradually over the next 32 h (Fig. 6b). The distribution of MB in suspension at 12 and 32 h were shown in Fig. 7. The proportion of degraded MB increased from 38.4% to 50.0%, whereas that of the adsorbed MB decreased from 56.3% to 47.6%, respectively. It could be observed that a small amount of MB dissolved in solution. Thus, the change of MB concentration in supernatant was the result of adsorption and degradation. This can be explained because the catalytic activity of nanocubes towards H_2_O_2_ decomposition was lower than that of nanorods, and a large number of H_2_O_2_ remained in the system (Fig. 6b). The high concentration of H_2_O_2_ in suspension could continuously provide the surface peroxide species, which caused the adsorption and degradation of MB over nanocubes.

The results suggested that the adsorption and degradation of MB was tightly related to the content of surface peroxide species via Ce^3+^-H_2_O_2_ interaction. A high concentration of H_2_O_2_ in the solution would enhance the adsorption and degradation of MB over the nanoceria surface. But the desorption of MB became dominant after the concentration of H_2_O_2_ was dramatically decreased.

### CR decolorization in nanoceria/H_2_O_2_ system

The decolorization process of CR in nanoceria/H_2_O_2_ system was also investigated at 25 °C. Control experiments showed that CR solution was stable in the presence of H_2_O_2_. The UV−vis spectral changes of the supernatant as a function of time were shown in Fig. S8. In presence of CeO_2_ nanorods, the intensity of the characteristic band of CR centered at 497 nm significantly decreased because a lot of CR adsorbed on the surface of nanorods. After the addition of H_2_O_2_, a quick and obvious increase (0 < t < 2 min) of the intensity of the characteristic band of CR was evident at the beginning of degradation. This feature was also observed for CeO_2_ nanocubes (Fig. S4b and S8). As previously reported, these features are mostly assigned to the desorption of CR from the surface of CeO_2_ because of the adsorption competition between CR and H_2_O_2_^10,13^.

**Figure 8:**
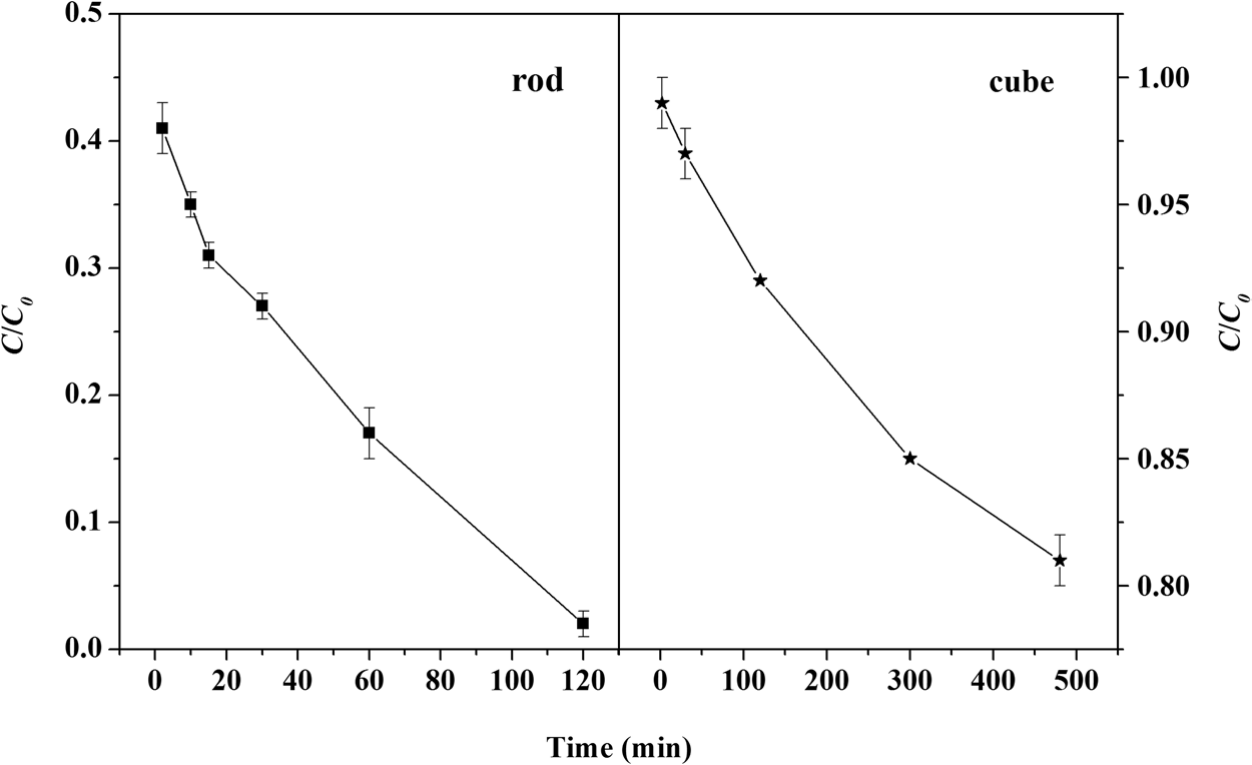
Remove of CR in nanoceria/H_2_O_2_ system (70 mg L^−1^ CR, 1.0 g L^−1^ CeO_2_, 20 mM H_2_O_2_).

Figure 8 presents the degradation kinetics of CR in nanoceria/H_2_O_2_ system. Obviously, CeO_2_ nanorods exhibited much higher catalytic activity for the degradation of CR than that of CeO_2_ nanocubes. 98% degradation of CR was achieved after 2 h of reaction time in nanorods/H_2_O_2_ system, while the degradation of CR was low, 8% after 2 h and 20% after 8 h, in nanocubes/H_2_O_2_ system (Figs S4c and S8). The high catalytic activity of the nanorods could be owing to its better redox properties and high concentration of Ce^3+^ ^5,6,11,46^. Furthermore, the higher adsorption capability of CR and the larger specific surface area was also conducive to CR degradation^40^. To further verify the importance of adsorption, a control experiment was tested. CeO_2_ nanorods was firstly mixed with 20 mM H_2_O_2_, and CR was added after several minutes. The degradation of CR was significantly decreased to 25.3%. This could be explained by the adsorption competition between CR and H_2_O_2_.

### Reusability of CeO_2_ nanorods

The recyclability of CeO_**2**_ nanorods was evaluated by CR degradation at the conditions of pH 9.0, 70 mg L^−1^ of CR, 20 mM of H_**2**_O_**2**_ and 1.0 g L^−1^ of CeO_**2**_. As seen in Fig. S9, the degradation percentage of CR almost kept unchanged during five successive runs after 2 h of reaction, indicating a good reusability of CeO_**2**_ nanorods.

### Reaction mechanism discussion

**Figure 9:**
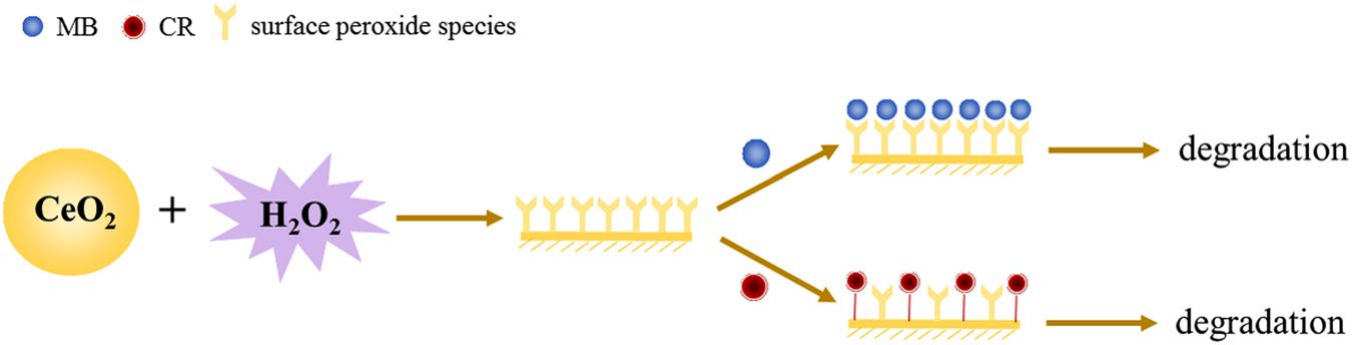
Schematic illustration for the possible mechanism of substrate-dependent Fenton-like activity of nanoceria.

In the nanoceria/H_2_O_2_ system, the nature of the oxidative species plays a key role in oxidative degradation of organic compounds. According to the literature^8–10,47,48^, Ce^3+^ on the surface of nanoceria could complex with H_2_O_2_ and generate surface peroxide species^13^, which would decompose into hydroxyl radicals at low pH or directly act as an oxidative species under alkaline conditions. We speculated that the surface peroxide species should also be the main oxidative species in the current nanoceria/H_2_O_2_ system because the experiments were performed at pH of 9.0. Based on the experimental observations, the adsorption-degradation processes of MB and CR are schematically illustrated in Fig. 9.

When MB was present in nanoceria/H_2_O_2_ system, MB could be adsorbed on the surface of nanoceria through electrostatic attraction with surface peroxide species, which subsequently act as the catalytic active sites for MB oxidation^47,48^. With respect to CR, the adsorption of CR on the nanoceria surface is through Lewis acid–base interactions, and the adsorption competition exists between CR and H_2_O_2_^13,14^. The degradation of CR would occur by attack from the adjacent peroxide species, which would induce an intermolecular rearrangement of CR^8,10^ or degrade CR through a homolysis of the O-O bond to form HO^5^.

Compared with CeO_2_ nanocubes, CeO_2_ nanorods displayed a higher catalytic activity for the decomposition of H_2_O_2_ and generated more surface peroxide species under the current conditions. Therefore, efficient degradation of CR was achieved in CeO_2_ nanorods/H_2_O_2_ system. However, MB could not be efficiently degraded by CeO_2_ nanorods in the presence of H_2_O_2_. The different chemical structure of the organic dyes may be one reason for the difference in the level of degradation because the degradation of MB is generally very difficult^14,49^. Furthermore, the high rate of H_2_O_2_ decomposition over the ceria nanorods would significantly decrease the concentration of surface peroxide species and the adsorbed MB, and hence the oxidation rate of MB.

## Conclusion

The adsorption-degradation of MB and CR in nanoceria/H_2_O_2_ system was investigated. The MB exhibited weak adsorption over the nanoceria surface via electrostatic attraction, while CR was adsorbed through Lewis acid–base interactions. An adsorption competition existed between CR and H_2_O_2_, whereas the adsorption of MB was enhanced by the formation of negative surface peroxide species through Ce^3+^–H_2_O_2_ interactions. The degradation of CR catalyzed by CeO_2_ was much faster than that of MB, and nanorods degraded the CR solution rapidly in comparison to that of nanocubes. In term of different adsorption behavior, two oxidation processes were suggested. MB is adsorbed over the surface of nanoceria through the interaction between MB and surface peroxide species, and the surface peroxide species will act as the catalytic active sites for oxidation of MB. But CR was adsorbed over nanoceria surface and attacked by the adjacent peroxide species, then oxidized into small molecules.

## Materials and Methods

### Materials

Ce(NO_3_)_3_·6H_2_O, NaOH, HCl, H_2_O_2_ (30%, w/w), MB and CR were purchased from Sinopharm Chemical Reagent Co. Ltd (Shanghai, China). All of the reagents were of analytical grade.

### Synthesis of CeO_2_ nanorods and CeO_2_ nanocubes

Nanoceria was synthesized through a hydrothermal process^30,37^. Typically, 40 mL of aqueous solution containing 0.05 g mL^−1^ Ce(NO_3_)_3_·6H_2_O and 0.15 g mL^−1^ NaOH was placed in a 50 mL Teflon-lined stainless-steel autoclave and heated. The CeO_2_ nanorods were synthesized by hydrothermal treatment at 120 °C for 12 h, and the CeO_2_ nanocubes were produced by hydrothermal treatment at 180 °C for 24 h.

### Catalyst characterization

The morphology and size of the nanoceria were determined by a high-resolution transmission electron microscope (HRTEM, JEOL, Japan). The powder X-ray diffraction (XRD) patterns were obtained on a D8 Advance X-ray diffractometer (Bruker, Germany). X-ray photoelectron spectra (XPS) measurements were performed on an ESCALAB 250Xi high-performance electron spectrometer (Thermo Fisher, USA). The Brunauer−Emmett−Teller (BET) surface area was measured by N_2_ adsorption-desorption isotherms recorded at 77.3 K (Quantachrome, USA). Raman spectra were recorded on a confocal microscopic Raman spectrometer (Renishaw In-Via, USA) with a 532 nm laser light irradiation from 100 to 1200 cm^−1^ at a duration time of 10 s. Before analysis, the samples with and without H_2_O_2_ treatment were pressed into slices.

The zeta potential of nanoceria as a function of pH was determined by the nanoparticle size and zeta potential analyzer (Horiba, Japan). The concentration of nanoceria was 20 mg L^−1^ and the pH of nanoceria suspensions were adjusted by 0.1 M HCl or 0.1 M NaOH.

### Decomposition of H_2_O_2_

The decomposition of H_2_O_2_ was carried out in a conical flask (250 mL) placed in a thermostat oscillator (Sunkun, China) with agitation at 200 rpm and 25 °C. 100 mg of the catalyst was added to 100 mL of 20 mM H_2_O_2_ solution (pH 9.0) as the beginning of the reaction. To avoid the influence of ions on the nanoceria activity, ultrapure water was used in the test, and the pH of the reaction system was adjusted using 0.1 M NaOH solution. At designated time intervals, a certain amount of suspension was taken out and filtered through a 0.22 μm membrane filter. The concentration of H_2_O_2_ was measured using an UV–vis spectrophotometer (Beijing Purkinje General, China) at 240 nm.

### Adsorption of dye in the absence of H_2_O_2_

The adsorption of the dye was performed in a 25 mL conical flask. Briefly, 10 mg of CeO_2_ powder in 10 mL of dye solution was ultrasonically dispersed and shaken at 25 °C. The pH value of the solution was fixed at 9.0, and the concentration of MB and CR was 15 mg L^−1^ and 70 mg L^−1^, respectively. After 30 min, the suspension solutions in the conical flasks were rapidly filtered through a 0.22 μm membrane filter, followed by immediate measurement of the dye concentration.

### Degradation of dye in nanoceria/H_2_O_2_ system

Generally, 100 mg of catalyst powder was added to 100 mL of dye solution at 25 °C, then H_2_O_2_ solution was added to reach a concentration of 20 mM and the concentration of dye in suspension was monitored. The initial concentration of MB and CR was 15 mg L^−1^ and 70 mg L^−1^, respectively. And the initial pH value of reaction system was 9.0. The concentration of MB and CR was measured using an UV–vis spectrophotometer (Beijing Purkinje General, China) at 664 nm and 497 nm, respectively. The all measurements were conducted in triplicate.

## Electronic supplementary material

Supplementary Material
