# Supplementary material for: Different adsorption-degradation behavior of methylene blue and Congo red in nanoceria/H2O2 system under alkaline conditions
Source: Sci Rep. 2019 Mar 21;9:4964. doi: 10.1038/s41598-018-36794-2 (PMC6428846; doi:10.1038/s41598-018-36794-2)
Supplement: Supplementary file 1 — Supplementary Material [file 41598_2018_36794_MOESM1_ESM.doc]

**Supplemental information for**

**Different adsorption-degradation behavior of methylene blue and Congo red in nanoceria/H_2_O_2_ system under alkaline conditions**

Xiaoshu Wei, Yi Wang, Yuqian Feng, Xiaomin Xie, Xiaofeng Li and Sen Yang*

Beijing Key Laboratory of Farmland Soil Pollution Prevention and Remediation, College of Resources and Environmental Sciences, China Agricultural University, Beijing 100193, China

*Corresponding author. E-mail: syang@cau.edu.cn. Tel/fax number: +86 10 62733470.


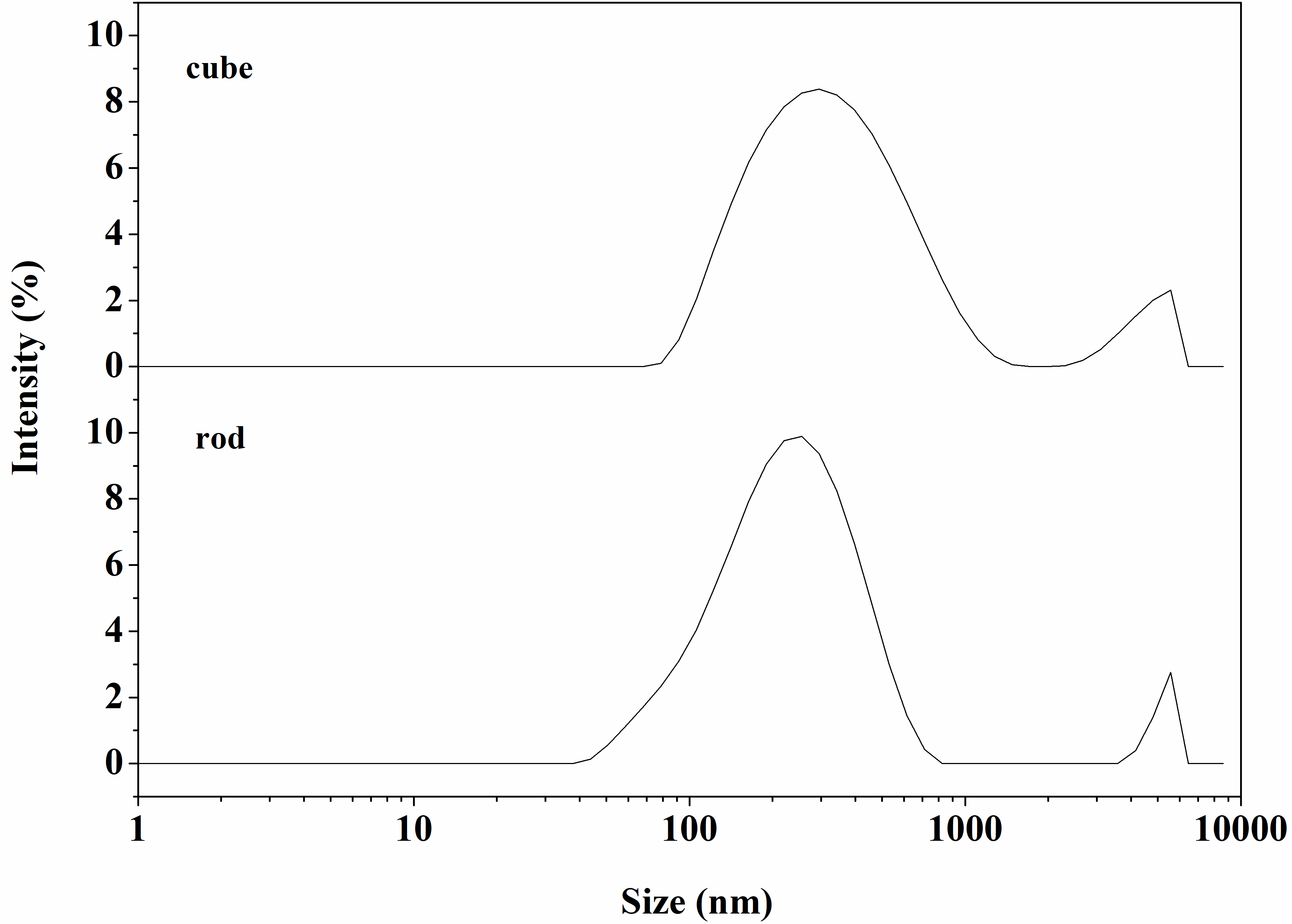


**Figure S1. Dynamic light scattering measurements of nanoceria.**


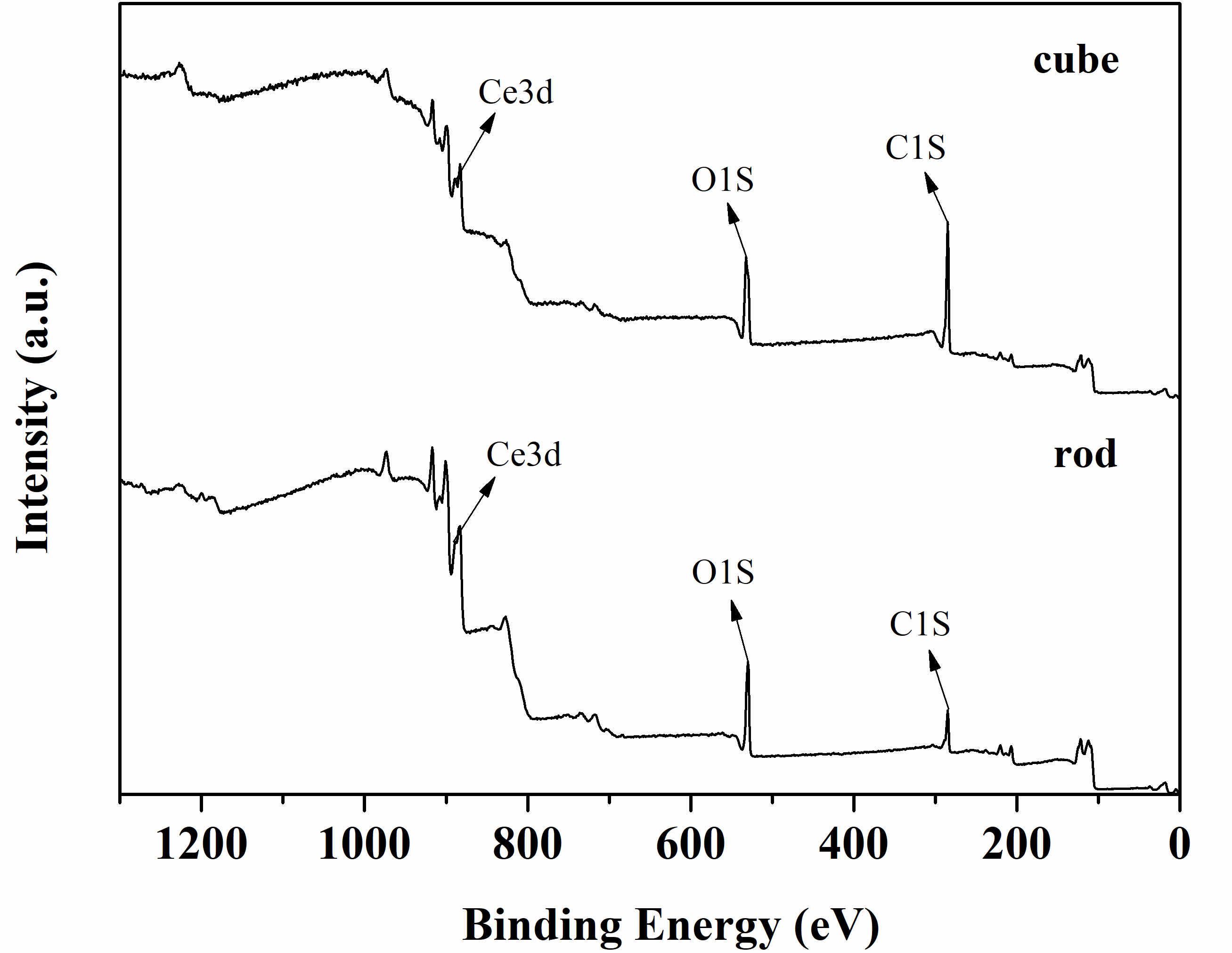


**Figure S2. XPS survey spectra of nanoceria.**


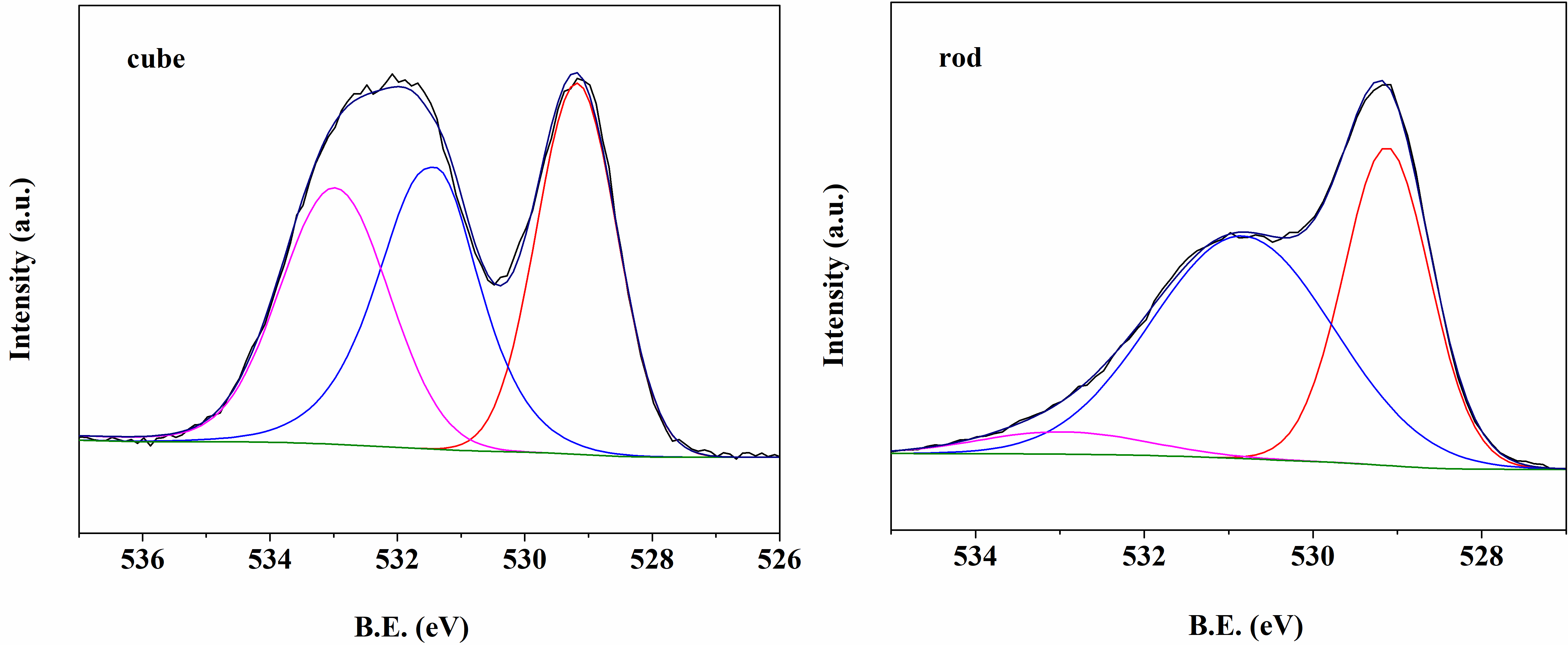


**Figure S3. O (*1s*) XPS spectra of of nanoceria.**


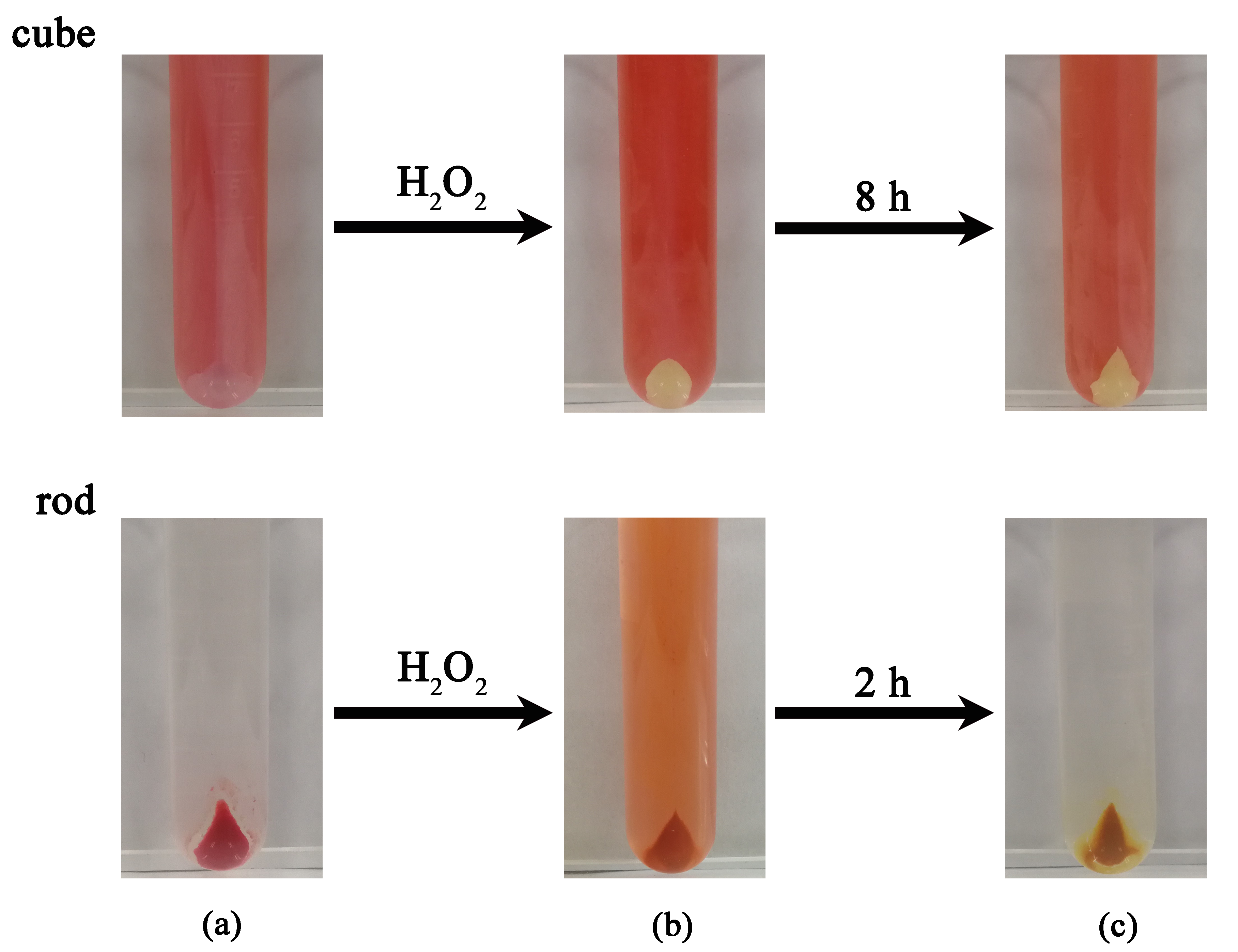


**Figure S4. The adsorption of CR on nanoceria surface (a); the adsorption competition between CR and H_2_O_2_ on nanoceria surface (b); and the degradation of CR in nanoceria/H_2_O_2_ system (c).**


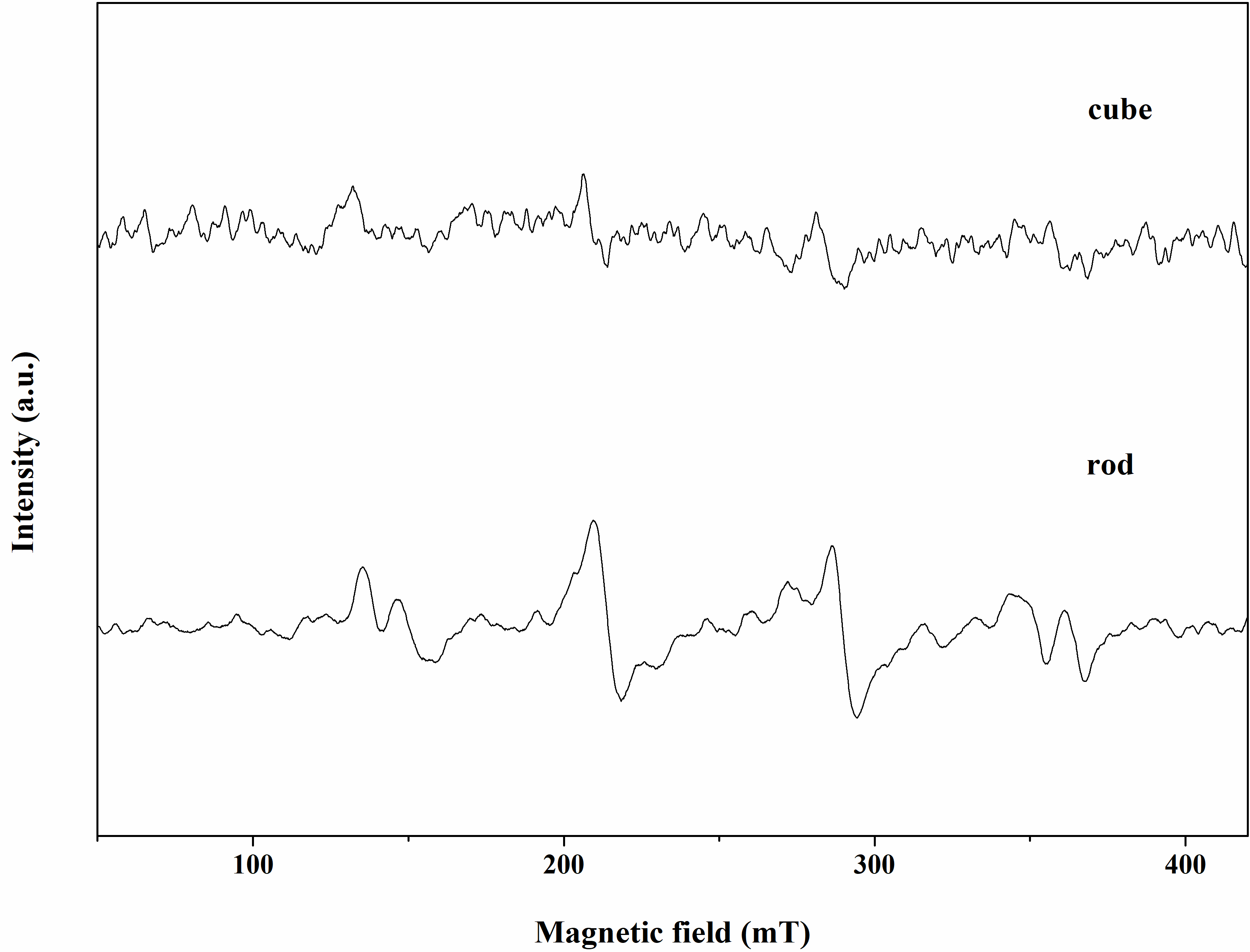


**Figure S5 DMPO spin trapping EPR spectra of CeO_2_/H_2_O_2_ system at pH 9.0. The incubation time is 2 min, and [CeO_2_] = 1.0 g L ^-1^, [H_2_O_2_] = 20 mM.**


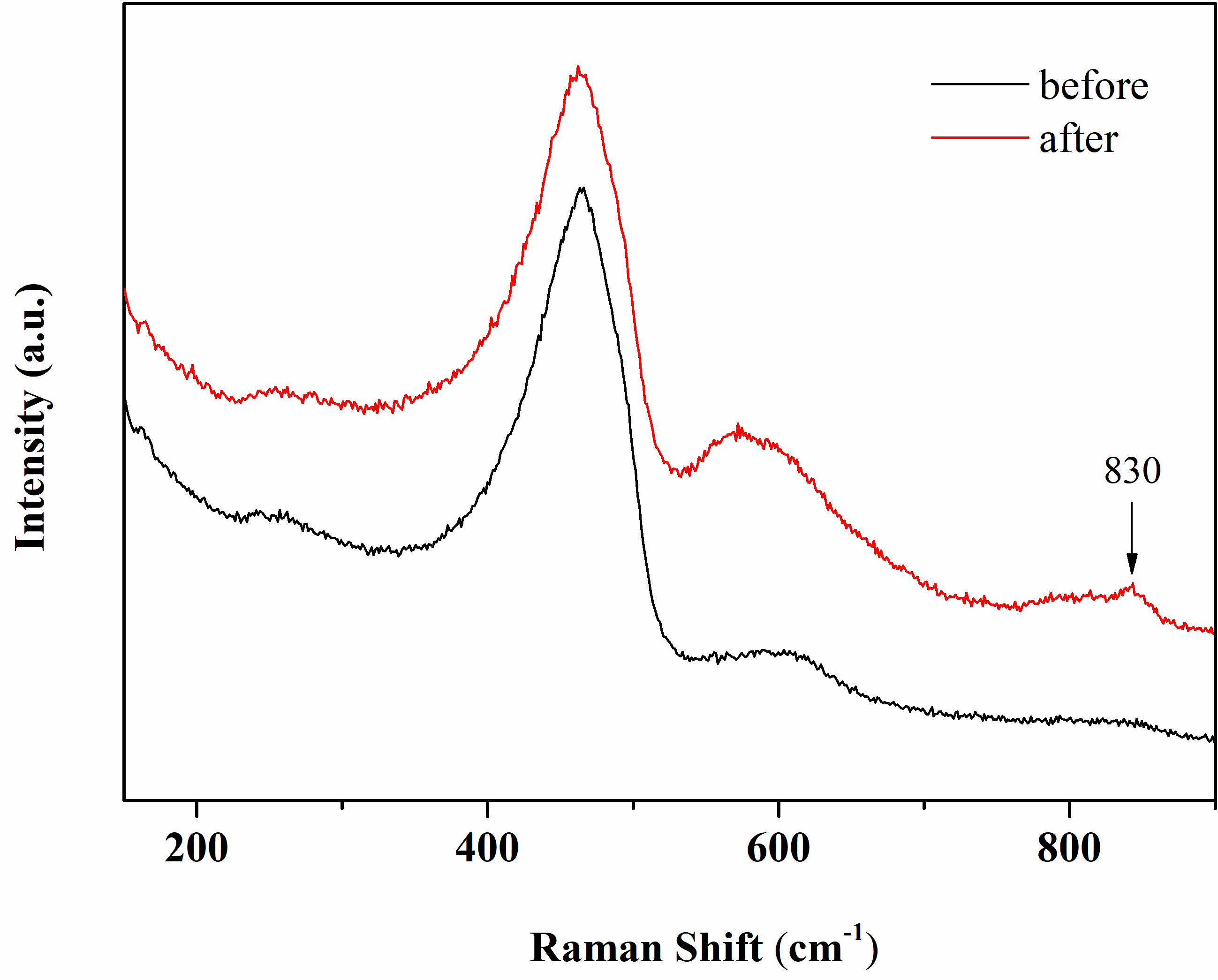


**Figure S6 Raman spectra of CeO_2_ nanorods before and after treatment with H_2_O_2_ at pH 9.0.**


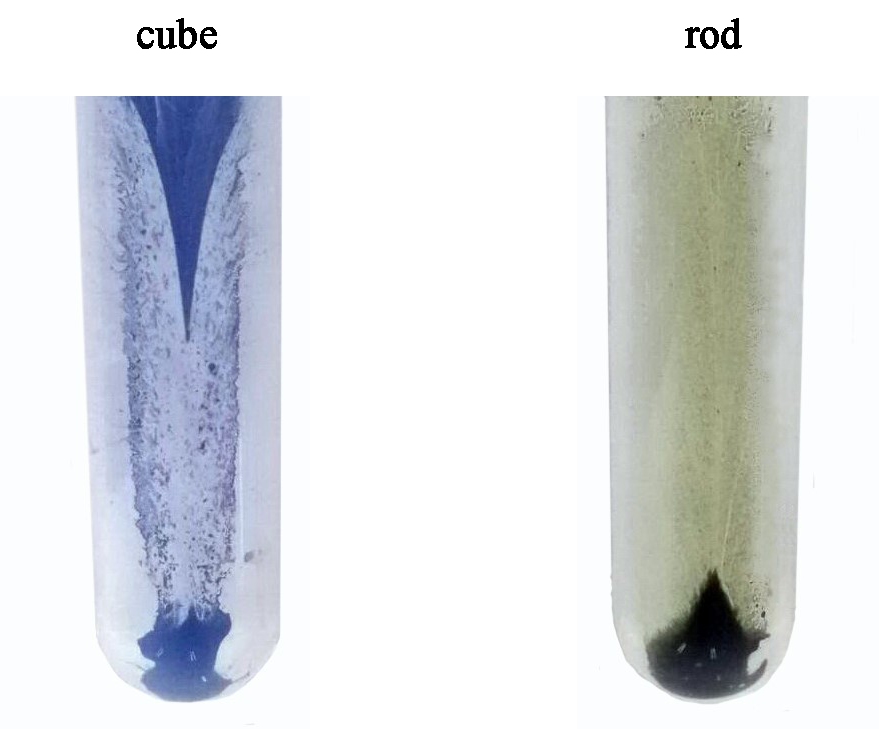


**Figure S7. The enhanced adsorption of MB on CeO_2_ nanocubes and nanorods in the presence of H_2_O_2_.**


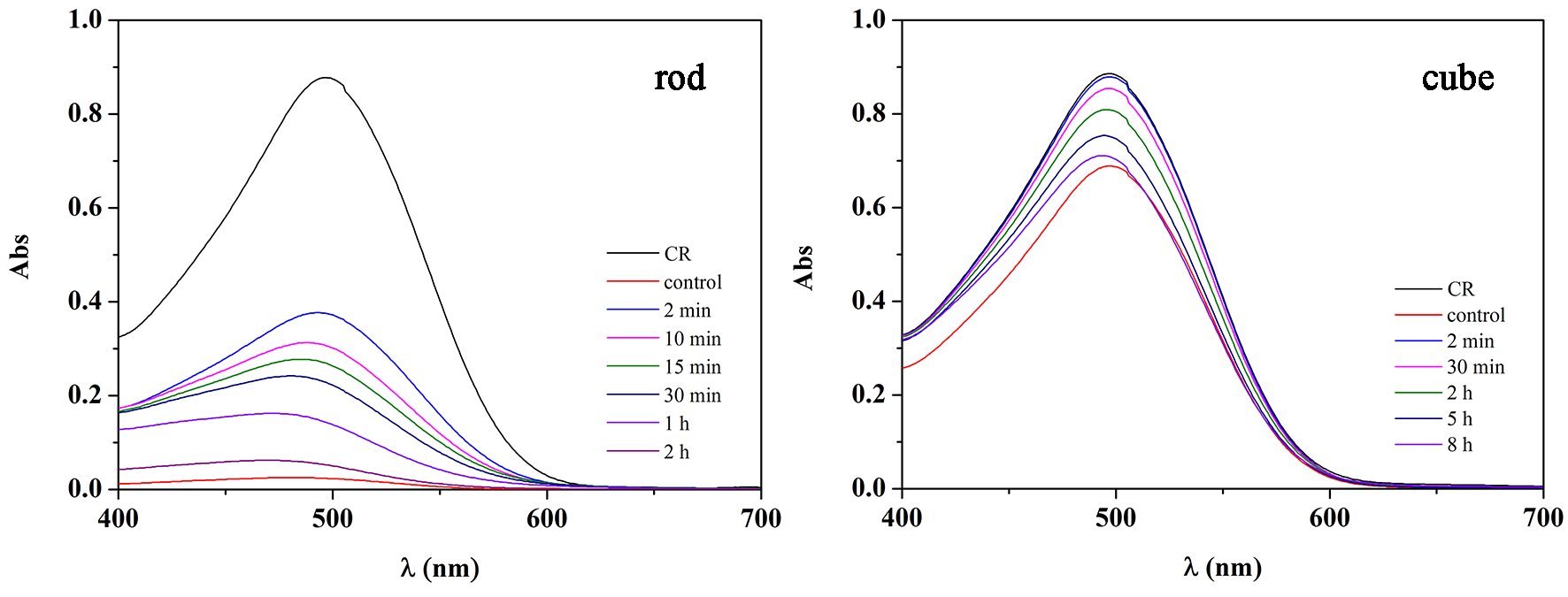


**Figure S8. Effect of contact time on CR decolorization in nanoceria/H_2_O_2_ system (70 mg L^-1^ CR, 1.0 g L^-1^ CeO_2_, 20 mM H_2_O_2_).**


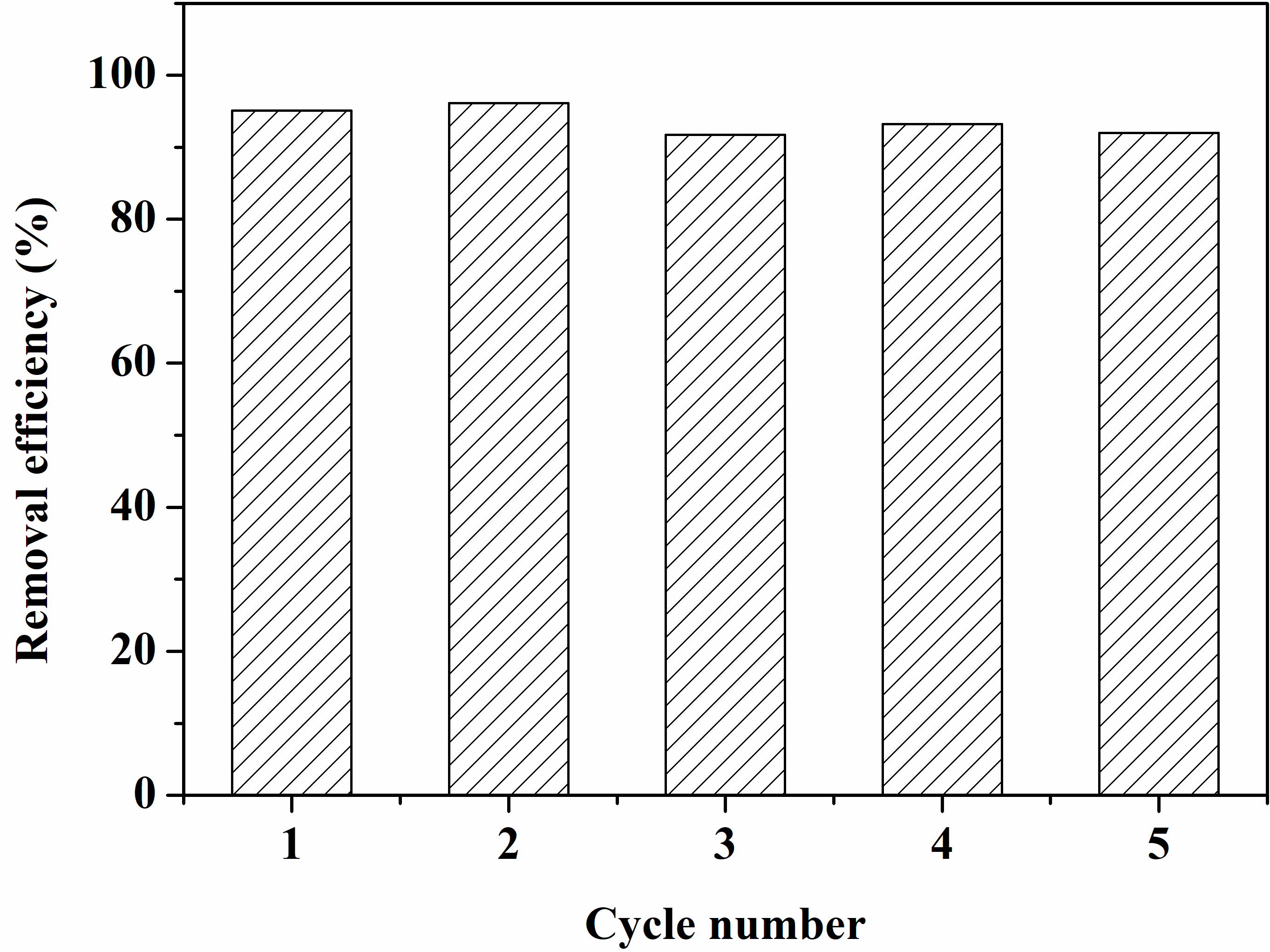


**Figure S9. Five consecutive experiments of the degradation of CR in nanoceria/H_2_O_2_ system (70 mg L^-1^ CR, 1.0 g L^-1^ CeO_2_, 20 mM H_2_O_2_, 2 h).**
